# Few-Shot Classification with Meta-Learning for Urban Infrastructure Monitoring Using Distributed Acoustic Sensing

**DOI:** 10.3390/s24010049

**Published:** 2023-12-21

**Authors:** Huynh Van Luong, Nikos Deligiannis, Roman Wilhelm, Bernd Drapp

**Affiliations:** 1 AP Sensing GmbH, Herrenberger Str. 130, 71034 Böblingen, Germany; roman.wilhelm@apsensing.com (R.W.); bernd.drapp@apsensing.com (B.D.); 2 Department of Electronics and Informatics, Vrije Universiteit Brussel, Pleinlaan 2, B-1050 Brussels, Belgium; ndeligia@etrovub.be; 3 Interuniversity Microelectronics Centre (IMEC), Kapeldreef 75, B-3001 Leuven, Belgium

**Keywords:** meta-learning, few-shot classification, distributed acoustic sensing, artificial intelligence, neural networks

## Abstract

This paper studies an advanced machine learning method, specifically few-shot classification with meta-learning, applied to distributed acoustic sensing (DAS) data. The study contributes two key aspects: (i) an investigation of different pre-processing methods for DAS data and (ii) the implementation of a neural network model based on meta-learning to learn a representation of the processed data. In the context of urban infrastructure monitoring, we develop a few-shot classification framework that classifies query samples with only a limited number of support samples. The model consists of an embedding network trained on a meta dataset for feature extraction and is followed by a classifier for performing few-shot classification. This research thoroughly explores three types of data pre-processing, that is, decomposed phase, power spectral density, and frequency energy band, as inputs to the neural network. Experimental results show the efficient learning capabilities of the embedding model when working with various pre-processed data, offering a range of pre-processing options. Furthermore, the results demonstrate outstanding few-shot classification performance across a large number of event classes, highlighting the framework’s potential for urban infrastructure monitoring applications.

## 1. Introduction

Fiber optic distributed acoustic sensing (DAS)—based on phase-sensitive optical time-domain reflectometry (ϕ-OTDR) [1,2]—is an emerging technology that detects acoustic signals and vibrations along tens of kilometers with high sensitivity and high data rates. DAS measures the strain change along an optical fiber by periodically injecting laser pules into the optical fiber and collecting the back-reflected Rayleigh scatter caused by small inhomogeneities along the fiber [3]. Essentially, the back-scattered light is used as an information carrier to infer parameters of the physical environment along the optical fiber. This technology is relevant to a large number of monitoring and surveillance applications, ranging from long-haul intrusion detection and structural health monitoring to the monitoring of railways, pipelines, and buildings [4]. The sensing principle offers several advantages over conventional point sensors, including temporally and spatially (quasi-)continuous measurements, remote monitoring in harsh environments, and robustness to electromagnetic inference. However, despite these advantageous attributes, DAS measurements also contain several challenges, primarily caused by dependence on external factors such as temperature and ground coupling and the high data rates involved (easily reaching terrabytes per day). Therefore, the efficient utilization of the data with the help of machine learning (ML) can significantly impact the effectiveness of DAS applications.

Deep Learning (DL) has emerged as a powerful paradigm for processing large numbers of unstructured data over the last decade [5]. For this reason, deep neural networks (DNNs) have recently seen growing adaptation for harnessing the extensive data generated by DAS systems: Aktas et al. [6] proposed to feed a five-layer convolutional neural network (CNN) with short-time Fourier-transformed (STFT) images for seismic event recognition; Shiloh et al. [7] proposed to use a 16-layer CNN (VGG16 [8]) for the task; and [9] proposed a convolutional long short-term memory (convLSTM) network, which combines a CNN for extracting spatial features from multi-channel signals and an RNN to analyze the temporal relationships over time. Peng et al. [10] achieved 94% prediction accuracy for seven different pipeline corrosion types based on a (deep) autoencoder in conjunction with a softmax layer.

One notable approach in [11] introduced a self-supervised DL method aimed at improving DAS measurements via mitigating spatially incoherent noise with unknown characteristics. In [12], different pre-processing methods for the input to various DL models were compared, and an accuracy of 99.2% in a four-way classification task was achieved, highlighting the importance of the initial data representation fed into a DL model. In [13], classical ML methods and DL methods were compared, and it was concluded that the best approach depends on the data regime: In the low-data setting (usually at a project’s beginning), classical ML approaches dominate DL methods, as neural networks tend to overfit on spurious correlations. In the high-data regime (usually at a later project stage), DL methods tend to outperform, as deep neural networks can discover unexpected patterns in the data that human experts may not have noticed.

In practice, the low-data regime is very common, though, as collecting a large labeled dataset is often both costly and time-consuming and sometimes infeasible due to operational constraints. Consequently, several works have explored different solutions to this challenge: In [7], a solution based on generative adversarial networks (GANs) was proposed. Leveraging GANs, their method augmented the training dataset for a supervised classifier using artificially generated DAS data. In another study [14], a purely unsupervised DL method was introduced, utilizing a convolutional autoencoder to extract features from DAS signals. Subsequently, a clustering algorithm identified the feature center of normal data, and the distance to the new signals was used to determine anomalies.

In this study, we focus on an urban monitoring application involving data collected from a 12.5 km long optical fiber in a metropolitan region. This area encompasses a variety of infrastructures, such as streets, road crossings, highways, and bridges. Our goal is to develop an ML framework to discern the normal operating conditions of these infrastructures from anomalies such as fatigue or damage, specifically in the low-data regime. For each specific asset, we create an event class to monitor its condition under normal circumstances, free from intrusions or anomalies. Anomalies, which include third-party intrusion (TPI) like machinery and manual shovel diggings, should be systematically detected and identified. The challenge here lies in multi-class classification while addressing the imbalanced training data issue, where certain classes have only limited training samples available. Moreover, our ML framework should be extendable and allow for the inclusion of new classes with minimal effort. Recent advancements in learning algorithms have demonstrated the potential of few-shot learning [15,16] for rapid adaptation to new tasks with limited labeled samples.

To address the aforementioned challenges, we introduce an ML framework leveraging few-shot learning through meta-learning [17] for classifying DAS data. Our approach involves the development of various pre-processing techniques for DAS data, with the resulting features being fed into a neural network to further refine the data representation. This representation then serves as the input for a few-shot classifier, which allows us to classify new query samples based on a limited number of support samples.

The remainder of this paper is structured as follows: Section 2.1 summarizes the used DAS system and configuration, Section 2.2 introduces the various pre-processing methods applied, and Section 2.3 provides a comprehensive overview of our few-shot classification with meta-learning framework. We subsequently evaluate the framework’s performance in Section 3, followed by the conclusion in Section 4.

## 2. Method

### 2.1. DAS System and Configuration

The DAS system [1,2,3] used in this study consists of a phase-sensitive DAS interrogator connected to an optical fiber acting as the acoustically sensitive element. The measurement process begins with the emission of a coherent light pulse into the optical fiber. As this pulse propagates through the fiber, it interacts with the impinging acoustic field. The scattered light, returned to the source due to Rayleigh scattering, carries information about the encountered perturbations. Acoustic signals induce variations in the optical path length, altering the backscattered light. The calculated phase signal of the backscattered light serves as an indicator of the acoustic or vibrational interactions. Consequently, the measured phase signal can be directly attributed to the acoustic or vibrational energy impinging on the fiber, facilitating accurate localization and characterization of events.

**Figure 1:**
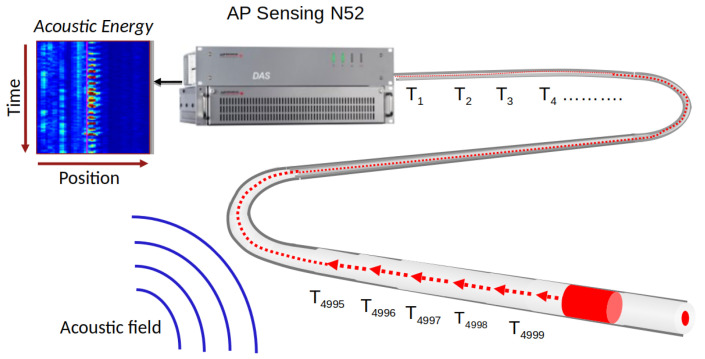
A phase-sensitive DAS diagram.

Figure 1 depicts a schematic diagram of a phase-sensitive DAS system employed in this study. To capture the acoustic signal patterns in the field, the sensor fiber is installed in an optical cable and laid in the ground. The measurement data are acquired by using an AP Sensing N52 DAS device, deployed in a metropolitan area. A sequence of coherent probe pulses is launched into the sensor fiber where events occur. Subsequently, the phase of the backscattered signal from the fiber is digitized and processed with the DAS device. Data are sampled at a repetition rate of 5000 Hz with a spatial resolution of 5 m.

### 2.2. Data Preparation

DAS data are obtained along an optical fiber and hence fundamentally have a two-dimensional structure comprising a time and a position axis. For each timestamp, a one-dimensional data array is generated along the sensing fiber, where the size of this data array is equivalent to the number of spatial sensing channels. These spatial channels of DAS maintain a repetition rate, serving as the sampling frequency for the detection signal.

We utilize various pre-processing methods to transform the phase data of DAS into valuable signal features, which are subsequently employed for training DNN models to learn a data representation. In our pursuit of exploring different time–frequency resolutions of the signal, we apply a discrete wavelet transform (DWT) [18] to decompose the phase data, taken every 60 s, from a specific spatial channel into distinct sub-bands. In this study, we employ the Daubechies-4 wavelet (db4) with four vanishing moments to facilitate precise time and frequency localization. Our chosen DWT is applied to decompose the phase data from each specific spatial channel into 4 sub-bands, specifically covering the frequency ranges of 0–16 Hz, 16–32 Hz, 32–64 Hz, and 64–128 Hz. The first type of feature is derived from the wavelet coefficients from these sub-bands for each channel, henceforth referred to as the decomposed phase (dePhase). The second set of features involves calculating the power spectral density (PSD) through the short-time Fourier transform (STFT) applied to the sub-bands. In addition, we introduce another set of features known as frequency band energy (FBE). These features are derived from PSD through the summation of the frequency dimension within each sub-band. It is worth noting that PSD includes an additional frequency dimension compared to FBE.

**Figure 2:**
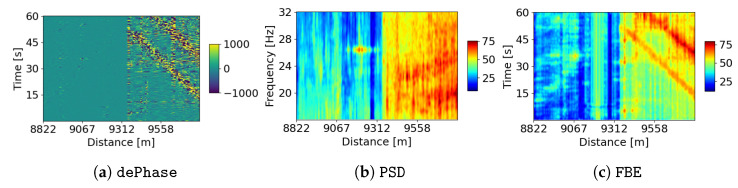
Various signal features—(**a**) dePhase, (**b**) PSD, and (**c**) FBE—are extracted from a fiber section within the 16 Hz–32 Hz frequency band.

Figure 2 depicts the three different types of extracted features within the decomposed frequency band of 16Hz-32Hz. Figure 2a displays 60 s of the decomposed phase plotted against distance. The power spectrum density, averaged over 60 s, is illustrated in Figure 2b, while Figure 2c presents the frequency band energy over 60 s. We can observe disturbances along the optical fiber using dePhase and FBE over time. It is worth noting that in Figure 2 higher scaled values are indicative of more pronounced disturbances. The same color scale for FBE is consistently applied throughout the entire paper. For instance, in the upper right corner of Figure 2a,c, we can observe a diagonal line representing a strong disturbance caused by a passing train. Furthermore, the PSD features offer insights into the energy distribution across the specific frequency range (16 Hz–32 Hz) for a particular position. More specifically, at approximately 9200 m, Figure 2b clearly illustrates a remarkable power density in the 26 Hz–27 Hz frequency range, which is characteristic of excavator engine noise. Distinctive patterns are also noticeable in Figure 2a,c around 9200 m, with regard to the dePhase and FBE responses over time. These representative features play a crucial role in training ML models, enabling them to learn patterns and interpret these learned patterns as the indicators of underlying events responsible for generating the disturbances.

### 2.3. Few-Shot Classification with Meta-Learning

We aim to develop an ML framework capable of learning insights into various events, enabling us not only to monitor the condition of infrastructure but also to detect intrusion events that may pose threats. As urban areas continually evolve, there arises a need to detect various forthcoming events, particularly those posing potential threats to infrastructure, such as machinery digging and cable theft. However, these new tasks involve a limited number of labeled samples due to the resource-intensive and impractical nature of collecting data. Few-shot learning through meta-learning [15,16] has emerged as a promising approach in the setting where ML models are trained on diverse learning tasks to tackle new tasks with minimal effort. In this work, we construct a framework for handling DAS data based on the architectures highlighted in [15,16]. This approach involves training an embedding model to learn a representation of multiple event classes, serving as a feature extractor, and a head classifier that is adapted to identify any new class on top of the learned representation.

**Figure 3:**
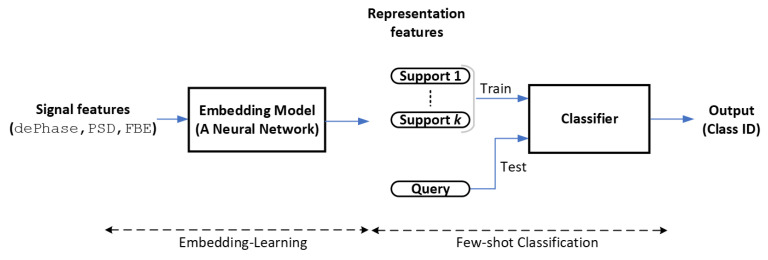
Few-shot classification framework of DAS data.

Figure 3 depicts our few-shot classification framework designed for DAS data. This framework is composed of two fundamental stages: the embedding-learning stage that employs a neural network as an embedding model and the few-shot classification stage that utilizes a classifier, such as a logistic regression or support vector machine [19].

#### 2.3.1. Representation Learning with an Embedding Model

The embedding model is trained for a multi-class classification task during the embedding-learning stage. We use Wide ResNet28 (WRN28) [20,21] as our embedding model for learning representations across different classes of transformed DAS data. Input features, including dePhase, PSD, or FBE, are fed into the WRN28 network to capture their representation features. It is important to remark that in this study, we examine the performance of each signal feature independently as it is fed into the embedding model. Our objective is to learn all signal responses required for monitoring infrastructure conditions along the fiber cable. Therefore, we categorize the signal features, as shown in Figure 2, into various classes. In this study, we simplify the labeling process by considering the events that happen every 500 m as one class, i.e., the 12.5 km cable length is equally divided into 25 sections as 25 distinct classes. This approach allows us to continuously monitor changes in infrastructure conditions over time, as we expect that the measured signals exhibit regularity under normal conditions.

**Figure 4:**
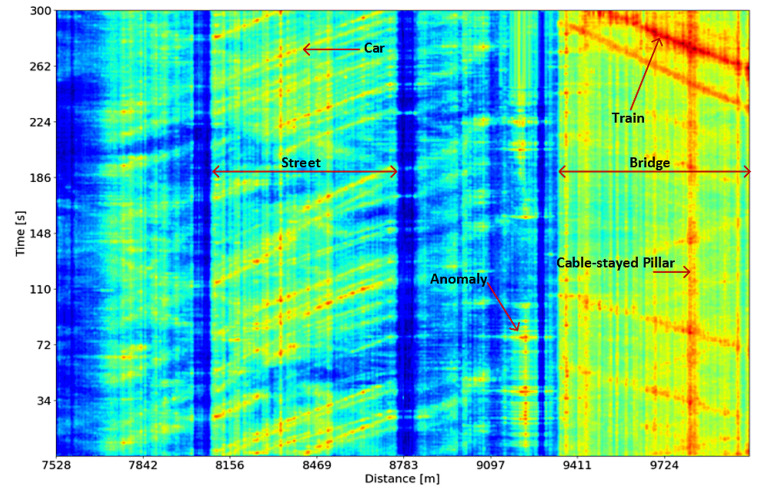
An FBE plot within a cable section showing various activities, including car, train, bridge, and cable-stayed pillar.

Figure 4 illustrates an FBE waterfall plot of some sections associated with various infrastructure types, e.g., street or bridge, which are excited by external actuators such as a car or train. Vibrations from the bridge can originate from specific components like a cable-stayed pillar. Additionally, the DAS system can also capture anomalies like TPI threats, e.g., mechanical and manual shovel diggings. Even though the trained embedding model has not been specifically trained on the anomalies data yet, the output features extracted via the embedding model can be fed into the classifier to categorize novel anomalies based on known support samples. This process is further elaborated upon in the subsequent section, which describes the few-shot classification stage of our approach.

#### 2.3.2. Few-Shot Classification

This stage aims at training a few-shot classifier to quickly add new tasks, even when provided with a limited number of labeled samples per class. The classifier should determine the class to which a new query sample belongs, utilizing a support set consisting of a small number of examples from the class. The classifier leverages the reuse of features extracted by the embedding model, which therefore plays a critical role in the classifier performance. A good embedding model yields high-quality representative features, ultimately contributing to superior few-shot classification performance. The classifier could be a simple model, e.g., a nearest-neighbor classifier, a logistic regression, a support vector machine [19], or a prototypical network [22]. In essence, the classifier can generalize to new classes, which are not encountered during the embedding-learning training, by effectively learning from a small number of support samples to make inferences about the query sample.

**Figure 5:**
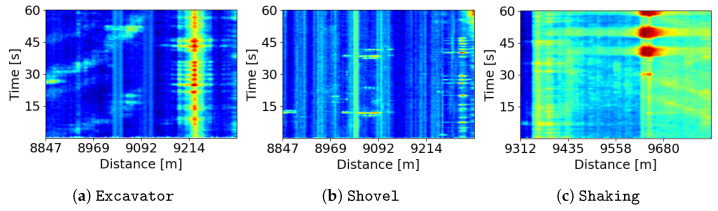
Examples of FBE plots illustrating anomalies in three new classes: (**a**) Excavator, (**b**) Shovel, and (**c**) Shaking.

Figure 5 presents three FBE plots of novel event classes representing anomalies that are not part of the initial training dataset. Specifically, Figure 5a,b illustrate instances of Excavator (at ∼9220 m) and Shovel (at ∼9290 m) diggings, respectively. These activities pose potential threats to critical infrastructure such as power cables and pipelines. Furthermore, as illustrated in Figure 5c, at a distance of ∼9620 m in the bridge section, we observe a Shaking disturbance that can potentially damage power or communication cables along the bridge. These new activities are not part of the embedding-learning training dataset; however, the classifier has the ability to adapt and classify them as new classes, provided some support training samples. Consequently, we can identify new event classes by updating the few-shot classifier without the need to retrain the embedding model.

## 3. Experiment

We conduct experiments using the dataset previously described. The embedding model is trained on a 25-class classification task, referred to as Meta-classes, and the few-shot classifier is subsequently tested on new tasks. Three types of signal features, the aforementioned dePhase, PSD, and FBE, are derived from the phase data. The data used for training the embedding model are collected over a four-month period without any anomalous events. Additionally, the phase data from several days featuring new events, such as Excavator digging, Shovel digging, and cable Shaking, are used to evaluate the few-shot classifier. The classifier leverages the representation features generated via the embedding model. In our work, we decompose the phase data into 4 bands every 60 s. To incorporate a broader context, we group 100 spatial channels together, resulting in each data sample containing 4 bands × 60 s × 100 channels of DAS data. PSD introduces an additional frequency dimension, achieved by applying the overlapped STFT with an FFT length of 128, yielding 65 frequency bins. In this study, we set the time resolution for PSD and FBE to 16 times per second.

**Figure 6:**
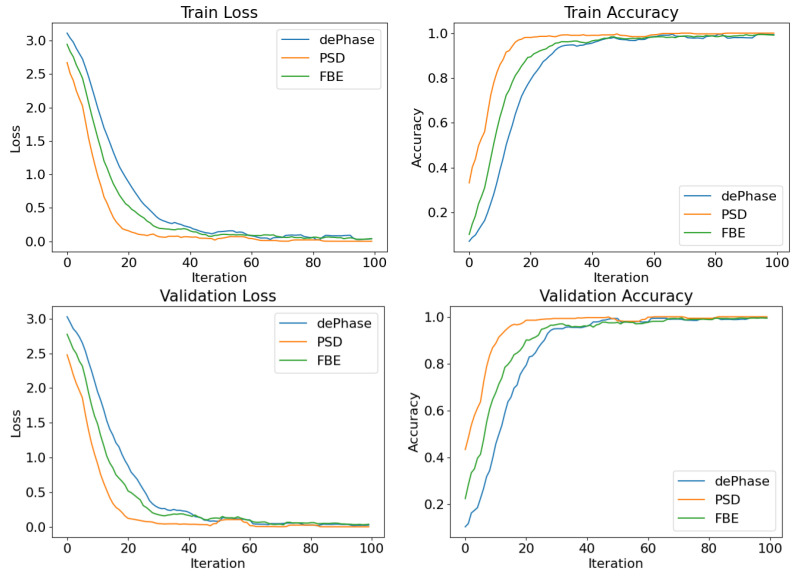
Loss and accuracy curves for embedding-learning on dePhase, PSD, and FBE datasets.

To assess the training progress of the embedding model, we plot the learning curves, including training and validation, for the embedding training across the three different features extracted from the DAS data. In Figure 6, the loss and accuracy curves are plotted against 100 learning iterations for dePhase, PSD, and FBE, respectively. The curves indicate that the embedding model with the PSD feature as input achieves faster convergence compared to the models using the dePhase and FBE features. Moreover, the learning curves associated with the PSD feature exhibit both the lowest converged loss and the highest converged accuracy. This suggests that the PSD signal features offer better information for training the embedding model.

The evaluation of the embedding model involves three metrics: Accuracy, Precision, and Recall on the Meta test set, including dePhase, PSD, and FBE, respectively.

- Accuracy quantifies the overall correct classification of the embedding model, representing the ratio of correct predictions to the total samples.
  (1) $$\mathrm{Accuracy}=\frac{\mathrm{Number}\mathrm{of}\mathrm{Correct}\mathrm{Samples}}{\mathrm{Total}\mathrm{Number}\mathrm{of}\mathrm{Samples}}$$
- Precision assesses the accuracy of positive predictions, indicating the proportion of correctly classified positive (True Positives) samples among those predicted positive samples (True and False Positives).
  (2) $$\mathrm{Precision}=\frac{\mathrm{True}\mathrm{Positives}}{\mathrm{True}\mathrm{Positives}+\mathrm{False}\mathrm{Positives}}$$
- Recall measures the completeness of positive predictions that is defined as the fraction of positive class samples (True Positives and False Negatives) that are correctly classified (True Positives).
  (3) $$\mathrm{Recall}=\frac{\mathrm{True}\mathrm{Positives}}{\mathrm{True}\mathrm{Positives}+\mathrm{False}\mathrm{Negatives}}$$

Table 1 reports the results of these three metrics for the multi-class classification performance of the embedding model on the Meta test set for each signal feature. We can observe that the performance of the test set on PSD gives the highest Accuracy, Precision, and Recall, followed by the performance of the test set on FBE and dePhase. The performance indicates that the embedding model delivers better performance with the frequency domain data, i.e., on PSD and FBE, in comparison to the performance with the phase data (dePhase).

We now evaluate the performance of the few-shot classifier across various few-shot classification tasks. Specifically, three types of anomalous activities are tested as new classes—examples of these new classes are shown in Figure 5. We employ a multinomial logistic regression, which is an extension of logistic regression designed to handle multi-class classification problems. This model utilizes the embedding features extracted from the output of the embedding model. We examine two settings, each with a limited number of support samples: one support sample (1-shot) and three support samples (3-shot). Additionally, we evaluate four combinations of tasks, as detailed in Table 2, which involves pairing each new task with the Meta-classes present in the embedding model’s training dataset. A set of classes, namely, TPI-classes, is created for evaluating the classifier performance exclusively with the new classes. Table 2 presents the few-shot classification performance achieved by the few-shot classifier. In general, the best performance is consistently observed for PSD, except in the case of the Shaking task with 3-shot, where the best result is achieved using FBE. It is noteworthy that the best results are highlighted in bold text in both Table 1 and Table 2. These results underscore the important role of the embedding model in achieving superior few-shot classification performance. Furthermore, when considering the number of support samples, the performance across all signal features with 3-shot consistently outperforms that with 1-shot.

## 4. Conclusions

We have introduced a few-shot classification framework for DAS data within the context of urban infrastructure monitoring, utilizing three different pre-processing methods, dePhase, PSD, and FBE, as the input data. The experimental results have demonstrated that the embedding model effectively learns the representation of DAS data across a large number of classes. These representation features significantly contribute to enhancing the classification performance of the few-shot classifier. We have conducted various experiments to evaluate both the embedding model and the classifier’s performance. The results show that the framework delivers outstanding few-shot classification performance, with the PSD features consistently outperforming FBE and dePhase. Moreover, high classification accuracy is achievable with either three or one support samples. Increasing the number of support samples leads to further enhancements across signal features. Our study consistently demonstrates superior performance with three support samples in comparison to one support sample.

## Figures and Tables

**Table 1:** Multi-class classification performance of the embedding model on the Meta test sets.

Metric [%]	dePhase	PSD	FBE
Accuracy	97.65	**98.85**	98.80
Precision	96.39	**98.13**	98.06
Recall	97.43	**98.36**	**98.36**

**Table 2:** Few-shot classification performance achieved by the few-shot classifier across various tasks.

Few-Shot Tasks	Metric [%]	dePhase		PSD		FBE	
		**1-Shot**	**3-Shot**	**1-Shot**	**3-Shot**	**1-Shot**	**3-Shot**
Excavator + Meta	Accuracy	95.70	96.18	**98.90**	**99.92**	97.97	99.20
	Precision	93.30	94.07	**97.88**	**99.82**	96.67	98.78
	Recall	94.85	95.56	**97.91**	**99.83**	97.26	98.82
Shovel + Meta	Accuracy	95.44	98.20	**98.60**	**99.72**	98.32	98.68
	Precision	92.78	97.30	96.70	**99.61**	**96.91**	97.46
	Recall	94.49	98.20	96.83	**99.63**	**97.07**	97.46
Shaking + Meta	Accuracy	93.20	94.68	**98.96**	98.84	98.04	**99.32**
	Precision	91.20	92.75	**98.06**	97.97	96.67	**99.22**
	Recall	92.75	94.40	**98.08**	97.99	97.08	**99.08**
TPI-classes	Accuracy	70.00	79.95	**92.42**	**93.91**	91.80	92.75
	Precision	70.06	78.05	**80.50**	**93.53**	78.36	87.82
	Recall	70.92	78.32	**80.71**	**93.65**	78.78	87.88

## Data Availability

Data are contained within the article.
